# Consecutive bilateral decompression retinopathy after mitomycin C trabeculectomy: a case report

**DOI:** 10.1186/s13256-016-0814-x

**Published:** 2016-02-04

**Authors:** Ana Raquel Marcos Figueiredo, Isabel Coutinho Sampaio, Maria João Fernandes dos Santos Menéres, George L. Spaeth

**Affiliations:** 1 Ophthalmology Department - Centro Hospital do Porto, EPE – Hospital de Santo António, Largo Professor Abel Salazar, 4099-001 Porto, Portugal; 2 Wills Eye Hospital, Jefferson Medical College, 840 Walnut Street, Philadelphia, PA 19107 USA

**Keywords:** Decompression retinopathy, Macular edema, Open angle glaucoma, Retinal hemorrhages, Trabeculectomy

## Abstract

**Background:**

After a successful trabeculectomy, a sudden intraocular pressure decrease may alter the intracranial to intraocular pressure ratio and cause decompression retinopathy. Frequent Valsalva maneuvers may also play a role in its pathogenesis. This condition may manifest as multiple retinal hemorrhages, edema of the optic disc, macular edema, or a sudden decrease in visual acuity postoperatively. Outcomes for patients are usually good, with spontaneous resolution occurring within a matter of weeks. It has been rarely reported in the literature as a bilateral condition.

**Case presentation:**

We present a case of consecutive bilateral decompression retinopathy in a 54-year-old severely obese Caucasian woman (body mass index 37 kg/m^2^) with open angle glaucoma and a poor history of medical therapeutic compliance, who chose surgical treatment based on her inability to consistently use ocular drops. Our patient underwent a trabeculectomy with mitomycin C in both eyes, with surgeries taking place 3 months apart. After the first surgery, 2 weeks postoperatively, she complained of decreased visual acuity. Examination of her right eye fundus revealed multiple retinal hemorrhages and disc edema. There was a similar pattern in her left eye, this time including maculopathy. Her visual acuity and fundoscopic changes resolved spontaneously over a period of a month in both cases. Currently, our patient has well-controlled bilateral intraocular pressure, ranging between 14 and 16 mmHg, without hypotensive medication.

**Conclusions:**

Decompression retinopathy is a potential complication after glaucoma surgery, but has rarely been described as a bilateral consecutive condition. A comprehensive approach could help to anticipate its occurrence and manage it.

## Background

Ocular decompression retinopathy was first described by Fechtner *et al*. in 1992 [1] as a complication of the abrupt iatrogenic lowering of intraocular pressure (IOP) after glaucoma filtering surgery. During intraocular surgery, entry into the eye allows the IOP to fall and equalize with the atmospheric pressure. If this change is sudden and large, it can induce hemodynamic changes that may result in bilateral decompression retinopathy. Immediately following surgery, the clinical situation is characterized by the appearance of diffuse retinal hemorrhages and edema of the optic disc in association with decreased visual acuity [2]. The most common optic nerve findings described are peripapillary and optic nerve head hemorrhages. Retinal manifestations mainly comprise intraretinal hemorrhages (92 % of retinal hemorrhages) and, less commonly, macular edema (3 %) or serous macular detachment (5 %) [13].

Outcomes are generally good for affected patients, with spontaneous resolution occurring within a few weeks. Over the years, the word “ocular” has been dropped, and “ocular decompression retinopathy” is now described in the literature as “decompression retinopathy” (DR). Besides a large drop in IOP after surgery, many of the cases presented in the literature have demonstrated a significant increase in IOP over a relatively short period of time pre-surgery, or large variations in IOP with spikes followed by significant drops in pressure [3]. These and other factors are believed to be related to the etiopathogenesis of DR [4]. Even the use of mitomycin C has been questioned as a potential adjuvant, and other hypotheses remain open [5].

There are few published cases of DR. Although Fechtner *et al*. described it as a complication following trabeculectomy (which represents half of total cases described), cases have been reported in other situations, such as neodymium: yttrium–aluminum–garnet iridotomies [6], anterior chamber paracentesis [7], pars plana vitrectomies [8], orbital decompression, and drainage implant insertions [9, 10]. In 2006, Bui *et al*. [11] were the first to describe maculopathy as an additional characteristic of DR. Our presented case concerns an extremely rare entity in ophthalmology, particularly with respect to our patient’s post trabeculectomy status, and represents an event rarely before described in the literature with this course and much less with a bilateral consecutive presentation.

## Case presentation

A 54-year-old Caucasian woman with open angle glaucoma and a history of suboptimal medical therapeutic compliance owing to an intolerance to drops was referred to our Ophthalmology Department. She was severely obese (body mass index 37 kg/m^2^) and had type 2 diabetes but was using insulin with good metabolic control. Her best corrected visual acuity (BCVA) in both eyes was 1.0. Her IOP was 35 mmHg without the use of medication, though she achieved values of 18 mmHg in her right eye and 16 mmHg in her left eye with the use of tafluprost once daily. No changes were identified in an examination of her anterior segment. The papillary cup was 0.3 in her right eye with a temporal notch and her left eye had a normal appearance.

Our patient’s compliance to medical therapy continued to be poor, because she blamed her drops for coughing and dyspnea attacks. Without the use of drops, her IOP values remained consistently in the 30s. After continuous non-compliance with several other ocular medications, surgery was discussed as an alternative treatment option. The risks and potential complications of surgery were explained to our patient. No signs of cornea or ocular media opacity, retinal hemorrhage, macular or peripheral detachments, or other contraindications were observed in either eye. Our patient decided to opt for surgery. Her right eye was operated on first with an uneventful mitomycin C trabeculectomy (0.3 mg/ml, 3 minutes).

**Fig. 1:**
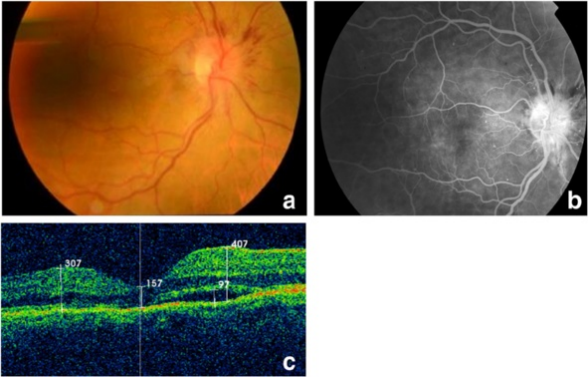
Right eye retinography, angiography, and optical coherence tomography 2 weeks after surgery. **a** Right eye fundus photograph showing multiple superficial, flame-shaped retinal hemorrhages located centrifugally from the optic disc associated with optic disc edema. **b** Fluorescein angiography exhibited macular microaneurysms associated with fluoroscein diffusion, peripapillary hemorrhages, and late optic disc leakage. **c** Optical coherence tomography image revealing folding of the macular retina and a small detachment of the neurosensory retina

On the first postoperative day, our patient presented with a diffuse, functioning, and non-leaking filtration bleb, associated with a well-formed anterior chamber, and an IOP of 8 mmHg, with a normal appearance on fundoscopy. Two weeks postoperatively she complained of decreased visual acuity; her BCVA was 20/32 and her IOP was 10 mmHg without medication. Fundoscopy exhibited multiple superficial, flame-shaped retinal hemorrhages located centrifugally from the optic disc associated with optic disc edema (Fig. 1a). There was no evidence of choroidal effusion. A fundus examination of her left eye was unremarkable.

**Fig. 2:**
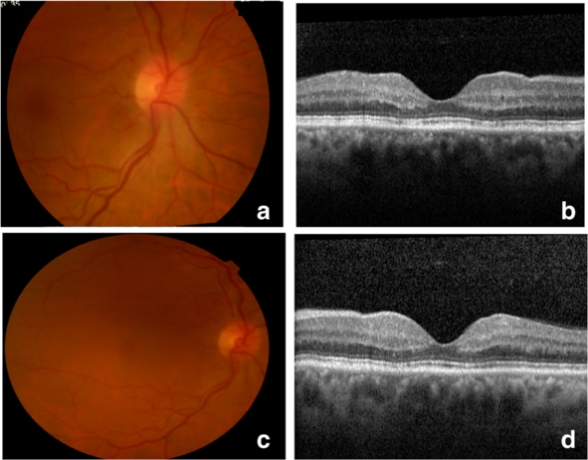
Right eye images 1 and 7 months after surgery. **a**, **b** Right eye fundus photograph and optical coherence tomography image 1 month after surgery. **c**, **d** Fundus photograph and optical coherence tomography image in the last follow-up visit (7 months after surgery). The peripapillary hemorrhages and optic disc edema spontaneously recovered

Optical coherence tomography (OCT) revealed folding of the macular retina associated with a small detachment of the neurosensory retina (Fig. 1c). The angiographic pattern showed macular microaneurysms associated with fluorescein diffusion, peripapillary hemorrhages, and late optic disc leakage, without ischemic areas or neovascularization (Fig. 1b). One month later the overall fundoscopic changes resolved spontaneously (Fig. 2a–d). Given this situation, a suspected diagnosis of DR was proposed. Other possible diagnoses were retinal venous occlusion or Valsalva retinopathy, but these were considered unlikely given the diagnostic results pattern. Subsequent follow-up visits were satisfactory, with our patient maintaining a steady IOP of 8–14 mmHg without medication. The peripapillary hemorrhages and optic disc edema spontaneously recovered during the second postoperative month, and our patient’s BCVA reached 20/25.

**Fig. 3:**
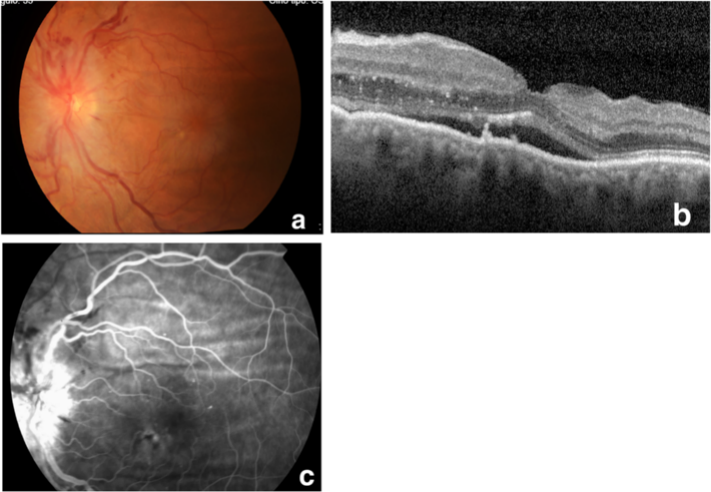
Left eye retinography, optical coherence tomography (OCT), and angiography 10 days after surgery. **a** Fundus photograph showing multiple peripapillary retinal hemorrhages, optic disc swelling, and macular edema. **b** Optical coherence tomography revealed macular edema with neurosensory retinal detachment. **c** Angiography demonstrated choroidal striation, optic disc leakage, and signs of macular microangiopathy and epitheliopathy

**Fig. 4:**
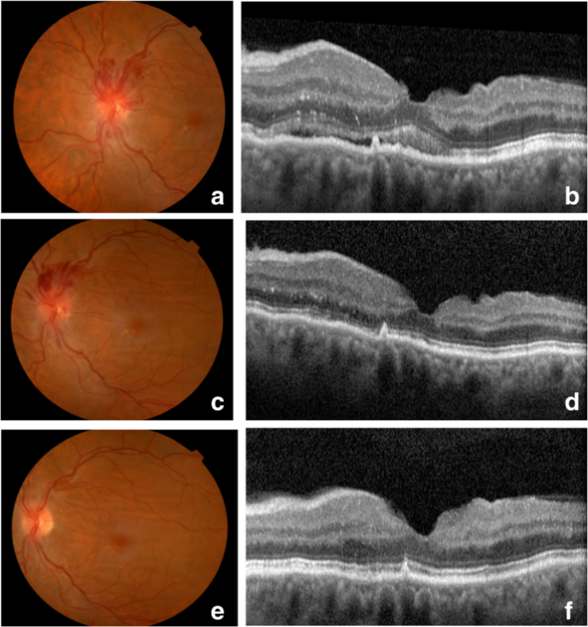
Left eye fundus photographs and optical coherence tomography image 2 and 4 months after surgery. **a**, **c** Gradual reduction of the localized hemorrhages and sectorial optic edema in the second postoperative month **b**, **d** Optical coherence tomography revealed progressive spontaneous macular edema recovery in the second postoperative month. **e**, **f** Images from the last visit (4 months after surgery)

Three months later, she underwent an uncomplicated left eye trabeculectomy with mitomycin C (0.3 mg/ml, 3 minutes). No postoperative hypotony was registered. On the first postoperative day, the anterior chamber was formed, the bleb was diffuse, her IOP was 8 mmHg, and results from fundoscopy were normal, without choroidals. The pattern of clinical evolution of this eye was similar, with decreased vision complaints 10 days after surgery (her BCVA was 20/32). Her IOP was 9 mmHg, with no medication. A fundus examination revealed identifiable multiple peripapillary retinal hemorrhages, optic disc swelling, and macular edema (Fig. 3a). OCT revealed macular folding and neurosensory retinal detachment (Fig. 3b). Choroidal striation, optic disc leakage, and signs of macular microangiopathy and epitheliopathy were visible on angiography (Fig. 3c). The localized hemorrhages and sectorial optic disc edema reduced progressively and her macular edema recovered spontaneously during the first postoperative month (Fig. 4). Her left eye IOP on the last follow-up visit was 16 mmHg without hypotensive medication and her BCVA was 20/25.

Given the occurrence of the same pattern in the contralateral eye, our patient’s complete medical history was reviewed again, with inquiries about possible hypertensive peaks, usual medication, and frequent Valsalva maneuvers. A summary infectious test was performed to rule out any possible systemic causes of bilateral papillitis. Results for the infectious study were negative.

## Discussion

Ocular DR presents as retinal hemorrhages and other fundoscopic changes following an acute lowering of IOP that cannot be explained by other processes [4]. The mean drop of IOP reported in the literature is 33.2 ± 15.8 mmHg (range, 4–57 mmHg) [12].

Our patient had all the described characteristics of DR, including retinal hemorrhages, optic disc edema, macular edema, and decreased visual acuity. Previous reports have described a subset of these characteristics in other cases. An article review revealed that DR resolves between 2 and 72 weeks (mean ± standard deviation of 13 ± 12.4 weeks), which is in agreement with what happened in our case [12]. Visual outcomes are generally good, with baseline vision returning in 85 % of cases. Previous studies have reported a mean drop in visual acuity from 20/50 to 20/100, though for most patients no intervention is required [12]. In our patient, her BCVA dropped from 20/20 to 20/25, which does not represent a significant decrease and can be considered a favorable outcome.

In previously reported cases, fluorescein angiography demonstrated normal retinal and choroidal vascular filling [12], which could help distinguish DR from other conditions associated with intraretinal hemorrhages. One such case was reported by Bui *et al*., where OCT scans exhibited macular edema and neurosensory macular detachment in the context of DR after trabeculectomy; however, this is not a common event in this context [11, 16, 17].

All of these findings alert us to the importance of a complete fundoscopic examination immediately after surgery in patients undergoing trabeculectomy or other hypotensive techniques involving low pressure, especially when pre-surgical pressure is high.

The main differential diagnosis for DR involves retinopathy associated with Valsalva maneuver or venous occlusion. Our patient’s clinical characteristics (that is, her physical constitution) could suggest the first diagnosis; however, typical ocular findings are classically described as pre-retinal hemorrhages with predilection for the macula and possible vitreous hemorrhages. Additionally, this condition is usually simultaneously bilateral. Venous occlusion was ruled out because there was no angiographic evidence of venous dilatation, delayed venous filling, or occlusive phenomena as is expected in acute central retinal vein occlusion. Furthermore, DR appears in patients who are typically younger, asymptomatic, and whose fundus examinations show a full return to pre-event state. A benign idiopathic intracranial hypertension pattern was also considered, given that our patient was severely obese. This possibility was rejected, by the absence of not only typical clinical findings (headache, nausea, and vomiting) but also optic disc edema on preoperative fundus examination. Additionally, her intracranial pressure was in the high-normal range, which matched her biotype [18]. Although carotid doppler ultrasonography was not performed, the lack of venous dilatation, ocular pain, peripheral retinal hemorrhages, and retina and optic disc neovessels, as well as her good visual recovery, make the diagnosis of ocular ischemic syndrome less likely. The diagnosis of DR was based on her typical history, retinal findings, and the exclusion of the other possible causes mentioned above.

Currently, there is no consensus about the etiologic mechanisms of DR, though mechanical and vascular origins are likely. One of the possible mechanical explanations is that the decrease in IOP into the low-normal range after trabeculectomy may alter the intracranial pressure/IOP ratio at the lamina cribrosa, resulting in its anterior shift and expansion [1, 19]. This forward movement might block axonal transport, leading to compression of the central retinal vein and producing optic disc hemorrhages and edema. This may explain why DR shares similar clinical findings with central retinal vein occlusion [1, 6, 12, 14, 20].

A likely vascular mechanism of DR is the loss of autoregulation of the retinal vessels due to longstanding glaucoma, which overwhelms their capacity to respond to changes in IOP (that is, reduced retinal arterial resistance causing an increased flow and leakage through fragile capillaries), resulting in retinal hemorrhage [1, 3, 7, 21]. Indeed, young individuals without hypertension or vasculopathy tolerate hemodynamic changes in choroidal vasculature very well. However, in patients in whom retinal vasculature autoregulation capacity might be impaired, DR can certainly occur.

Interestingly, the fact that DR presented bilaterally probably denotes individual susceptibility, as it is based on vascular instability and pressure fluctuations.

To the best of our knowledge, this is the first case of consecutive bilateral DR after mitomycin C trabeculectomies in the context of open-angle glaucoma. This discussion on the mechanisms behind its appearance may help predict the occurrence of DR and thus suggest other surgical modalities for patients with glaucoma who are potentially at risk.

## Conclusion

DR is an exclusion diagnosis. Impaired autoregulation of the retinal vasculature, in which acute lowering of the IOP increases blood flow through the retinal capillary bed, leads to multiple focal leaks presenting as blot hemorrhages. Valsalva maneuver, transitory hypotony, and mechanical factors may play a role, either in combination or individually, in the pathogenesis of DR.

Fundoscopy reveals a multifocal hemorrhagic retinopathy possibly associated with optic disc or macular changes. Generally, the overall fundoscopic changes gradually fade without sequelae.

Although DR infrequently results in significant ocular morbidity, a gradual reduction in IOP might prevent this complication [12]. Nevertheless, if a rapid reduction in IOP is critical to preserving vision, this goal should be pursued because DR tends to have a benign course with visual acuity returning to preoperative levels without any treatment.

Although DR is a potential complication after glaucoma surgery, we are unaware of other published and individually discussed clinical case reports of a consecutive bilateral presentation.

## Consent

Written informed consent was obtained from the patient for publication of this case report and accompanying images. A copy of the written consent is available for review by the Editor-in-Chief of this journal.

## Abbreviations

BCVA: Best corrected visual acuity
DR: Decompression retinopathy
IOP: Intraocular pressure
OCT: Optical coherence tomography
